# Constitution and By-Laws of the Central States Dental Association

**Published:** 1866-07

**Authors:** 


### Proceedings of Societies.

### CONSTITUTION AND BY-LAWS OF THE CENTRAL STATES DENTAL ASSOCIATION.

### Article I.—Name.

This Society shall be known as The Central States Dental Association.

### Article II.—Objects.

The objects of this Association shall be, to cultivate the science and art of Dentistry and all its collateral branches, to elevate and sustain the professional character of Dentists, to promote among them a mutual improvement, social intercourse and good feeling, and to collectively represent and have cognizance of the common interest of the dental profession in the Central States.

### Article III.—Members.

Active members shall consist of regular dental practitioners. Physicians and dentists not regularly in the dental practice may become honorary members.

### Article IV.—Qualifications for Membership.

1. Good moral character: evidence to be presented by two reputable persons.

2. Honorable, manly, and friendly feelings towards the members of the profession.

3. A determination to practice dentistry as an art founded upon science, or as a liberal profession.

4. Must be considered a reputable practitioner of dental surgery by the members of the profession who are acquainted with him.

5. Must be a graduate of a Dental College or a graduate of a Medical College, and for one year a regular practitioner of dentistry ; or if not a graduate, must have been a respectable practitioner of dentistry for at least six years; and if the candidate commences the practice after this date, he must invariably be a graduate.

### Article V.—Election of Members.

1. Applicants for membership shall present their name and place of residence, in writing, to the Secretary, accompanied with a fee of one dollar, who will refer their application to the Committee on membership, and, if elected, shall sign the Constitution before they can take part in the proceedings.

2. Any reputable practitioner of medicine may become an honorary member of this society.

3. An affirmative vote of two-thirds of all the members present shall be necessary to an election of a new member.

### Article VI.—Election of Officers.

The officers shall be elected by ballot, a majority of all the votes cast being necessary to a choice. The committees to be appointed by the President.

### Article VII.—Officers.

The officers shall consist of a President and three (3) Vice Presidents, Recording and Corresponding Secretary, Treasurer and two Standing Committees, viz : an Executive, and a Committee on Membership, who shall serve for one year, or until their successors are duly elected.

### Article VIII.—Duties of Officers.

1. It shall be the duty of the President to preside at all the meetings, and, with the Vice President and Secretary, to call extra sessions when occasion may require it.

3. It shall be the duty of the senior Vice President present to preside in the absence of the President, and to perform the duties devolving upon that officer, and also to counsel with the President in difficult cases.

4. The Recording Secretary shall keep a record of the proceedings of the society, take charge of all books, papers and documents belonging to the society and file the same, and serve notices.

5. It shall be the duty of the Corresponding Secretary to conduct the correspondence of the society not connected with its members, to keep a copy of all letters written by him, and a file of all he may receive. He shall report to the society as often as anything comes to him officially, or of interest to the association.

6. It shall be the duty of the Treasurer of the society to hold in trust any and all funds belonging to the society, and pay them out on the order of the Secretary; and to collect all assessments.

### Article IX.—Duties of Committees.

1. It shall be the duty of the Executive Committee to arrange subjects for discussion at least two months before each meeting and furnish them to the Secretary for publication; to secure and prepare a suitable room for the holding of the meetings, and to perform such other duties as the President may direct.

2. It shall be the duty of the Committee on Membership to thoroughly examine into the qualifications of applicants in regard to moral character and practical dentistry, and recommend their admission or rejection or refer their case to the Association.

### Article X.—Of Students.

No member of this Society shall receive a student for a shorter period than two years unless the student has already received instructions to equal that term, and the student agreeing to attend two full courses of lectures, the last of which shall be in a regular dental college.

### Article XI.—Meetings.

1. This Association shall hold two meetings a year. The annual meeting shall be held in the city of Louisville, Ky., during the Christmas holidays, and the semi-annual meeting to be held on the second Tuesday in April, the place of meeting to be determined at the annual meeting.

2. Nine active members shall constitute a quorum for the transaction of business.

ARTICLE XII.—AMENDMENT.

This Constitution may be amended by the proposition to so amend being filed with the Secretary one year, and published with the proceedings of the Society.

NAMES AND RESIDENCES OF MEMBERS.

Dr. W. H. Morgan, Nashville, Tenn.
" W. M. Rogers, Shelbyville, Ky.
" J. A. McClelland, Louisville, Ky.
" S. Griffith, " "
" W. D .Stone, " "
" W. H. Shadoan, " "
" W. G. Redman, " "
" G. B. Fittz, " "
" C. E. Dunn, " "
" L. E. Wilson, St. Louis, Mo.
" J. H. Bedford, Louisville Ky.
" John Ragland, Nashville, Tenn.
" S. Driggs, Lexington, Ky.
" G. S. Jones, Russelville Ky.
" J. W. Baxter, Warsaw, Ky.
" R. H. Wilson, Louisville, Ky.
" B. M. Gildea, " "
" S. J. Cobb, Nashville, Tenn.
" J. F. Canine, Louisville, Ky.
" R. Peckover, Paris, Ky.
" L. D. Walter, Cincinnati, Ohio,
" G. W. Field, " "
" J. L. Nourse, Cloverport, Ky.
" G. F. Lampkin, Louisville, Ky.
" W. H. Gates, " "

Dr. G. W. Monroe, Fishersville, Ky.
Prof. J. Taft, Cincinnati, O.
Dr. J. M. Buckley, Nashville, Tenn.
" J. T. Noel, Galatin, Tenn.
" L. T. Gunn, Nashville, Tenn.
" E. Q. Naghel, New Albany, Ind.
" Thos. Walch, Murfreesboro, Tenn.
" T. E. Beech, Franklin, Tenn.
" J. H. Floor, Lexington, Ky.
" W. F. Southern,
" Alex. Hartman, Murfreesboro, Tenn.
" F. W. Garkey, Memphis, Tenn.
" C. P. Baird, Winchester, "
" R. Russell, Nashville, "
" John Fouche, Knoxville, "
" J. C. Ross, Nashville, "

### BY-LAWS.

Honorary members shall be entitled to seats at all meetings of the Society, and shall have the privilege of participating in all discussions not involving pecuniary expenditures.

### Order of Business.

1. Calling Roll of Officers.
2. Calling Roll of Members.
3. Reading Constitution and By-Laws.
4. Reading minutes.
5. Reports of Officers and Committees.
6. Election of new members.
7. Election of officers.
8. Reception of members not present at opening of the meeting, and reading notes from absentees.

SECOND DAY.

9. Selection of place for semi annual meeting, to take place at 10 o'clock, A. M.

10. Appo'ntment of Standing Committees.

11. Unfinished and miscellaneous business.

12. Adjournment.

ARTICLE 3.—OF PRIVILEGES, ETC.

No member shall be permitted to speak longer than ten minutes, or more than once until all have had an opportunity to speak, and not more than twice on any one subject unless by express permission of the Society.

ARTICLE 1.—CONDUCT OF MEMBERS.

Any act of special immorality or unprofessional conduct committed by a member of this Association, shall be referred to the Committee on Membership, whose duty it shall be to thoroughly examine into the case and report at the next regular meeting if the charges be sustained. Whereupon by vote the offending member may be reprimanded or expelled; a two-thirds vote being necessary for expulsion, a plurality being sufficient for a reprimand.

ARTICLE 5.—CONTRIBUTIONS.

This Society may receive contributions in money, books, or other property which may be used or disposed of in furthering the objects of the Society.

ARTICLE 6.—LIMITATION OF MEMBERSHIP.

Any member failing to pay his dues for two successive years shall be dropped from the roll of membership.

ARTICLE 7.—AMENDMENT.

These By-Laws may be amended by the proposition being filed with the Secretary one year, and receiving a vote of two-thirds of all the members present,

